# Outlines of Modern Chemistry, Organic

**Published:** 1877-10

**Authors:** 


### BIBLIOGRAPHICAL.

OUTLINES OF MODERN CHEMISTRY, ORGANIC—Based in part upon Riche's Manuel de Chimie. By C. Gilbert Wheeler, Professor of Chemistry, in the University of Chicago, etc. Chicago: Janson, McLany & Co., 1877.

Time has not been allowed for such perusal of this work as is necessary to give an opinion of its merits. Two hundred pages are occupied in the principles of organic, to the exclusion of those involved in the study of inorganic and general chemistry. It is intended for the use of students, and is supposed to be suited to their wants, considering the limited extent to which the subject has attracted interest.

